# Trends and Associated Factors of Hypertension among Residents Aged ≥15 Years in Guangzhou, China, 2004–2013

**Published:** 2018-02

**Authors:** Xiaomei DONG, Jiaming RAO, Yunfeng YE, Minhui PANG, Jianwei YANG, Haiqing WANG, Jiayi ZHANG, Bingying PAN, Xueji WU, Xiongfei CHEN

**Affiliations:** 1. Center for Injury Prevention and Control, School of Medicine, Jinan University, Guangzhou, 510632, China; 2. Shenzhen Baoan Center for Chronic Diseases Prevention and Treatment, Shenzhen, Guangdong, 518100, China; 3. Guangzhou Center for Disease Control and Prevention, Guangzhou, 510440, China

**Keywords:** Hypertension, Trend, Prevalence, China, Risk factors

## Abstract

**Background:**

We aimed to describe the trends and associated factors of hypertension among residents aged ≥15 yr in Guangzhou, China.

**Methods:**

Three standardized cross-sectional health surveys were conducted in 2004, 2009 and 2013 using a multi-stage cluster sampling method, and a total of 69128 qualified participants were included in the study. The data were obtained through physical health examination and questionnaire survey.

**Results:**

The age-standardised prevalence of hypertension increased from 12.5% to 16.0% between 2004 and 2009 and declined from 16.0% to 14.0% between 2009 and 2013, and crude prevalence respectively was 14.6%, 19.1% and 18.8% in 2004, 2009 and 2013. The proportion of optimal blood pressure dropped from 51.1% to 33.2%, high-normal blood pressure increased from 20.1% to 28.9%, grade 1 hypertension and grade 2 or 3 hypertension increased from 11.5% to 13.6% and 3.9% to 5.8% between 2004 and 2013. The average age was significantly increased (*P*<0.001) from 42.8 to 47.5 yr, and the average body mass index slightly increased (*P*<0.001) from 22.4 to 23.0. Logistic regression analysis shows that higher age, male, higher body mass index, smoking and drinking alcohol were potential risk factors for hypertension.

**Conclusion:**

Both crude and age-standardized prevalence of hypertension were initially increased, but subsequently decreased in Guangzhou during 2004–2013. The optimal blood pressure population decreased significantly while the high-normal blood pressure population increased substantially during the survey period.

## Introduction

Hypertension is the main cause of mortality in China as well as worldwide because of its high frequency and major risk of cardiovascular disease (1,2). The hypertension count will rise to 1.56 billion people by 2025 (3). According to a national hypertension survey of China, the prevalence of hypertension in adults was 18.8% or an estimated 200 million people, which accounts for approximately one-fifth of the total global incidences (4). At least 7.60 million people worldwide die from hypertension-associated cardiovascular diseases annually, which accounts for 13.5% of all-cause mortality (5). Hypertension is an independent risk factor for several cardiovascular events, such as stroke, sudden death, heart failure and peripheral artery disease (6,7). The prevention and treatment of hypertension are important for reducing the incidence and mortality of cardiovascular disease. Hypertension including non-modifiable factors such as age, gender, genetic factors, as well as modifiable factors including overweight, high sodium intake, smoking, alcohol consumption, and reduced physical activity (8), we need to change these modifiable factors to decrease blood pressure (BP) and even prevent the development of hypertension.

In this study, hypertension was defined an average systolic BP (SBP) ≥140 mmHg, and/or an average diastolic BP (DBP) ≥90 mmHg, or currently taking antihypertensive drugs (self-reported). Definitions and classifications of BP levels (mmHg) in our analysis were based on the 2007 Guidelines for the Management of Arterial Hypertension (9). Namely, optimal BP was defined as <120/80 mmHg, normal BP as SBP 120–129 and/or DBP 80–84, high-normal BP (prehypertension) as SBP 130–139 and/or DBP 85–89, grade 1 hypertension as SBP 140–159 and/or DBP 90–99, grade 2 hypertension as SBP 160–179 and/or DBP 100–109, and grade 3 hypertension as SBP ≥180 mmHg and/or DBP ≥110 mmHg. Grade 2 hypertension and grade 3 hypertension were combined into 1 group in this study because of the relatively smaller sample size in both groups. Body mass index (BMI) was calculated as weight (kg) divided by height (m) squared, and was categorised as underweight (<18.5 kg/m^2^), normal weight (18.5–23.9 kg/m^2^), overweight (24.0–27.9 kg/m^2^) or obese (≥28 kg/m^2^).

Guangzhou is the provincial capital of Guangdong province, which is in southern China, and had a population of 1.6 million in 2015. Its gross domestic product rapidly grew from 44.5 million Yuan (2004) to 167 million Yuan (2014), with an average growth rate of 9.7% over the last decade (10). With the world’s fastest economic and urban development, Guangzhou provides an ideal platform to measure the trends in hypertension prevalence. Hypertension prevalence in Guangzhou had already reached a high in 2008 (11), but trend data were lacking. This study used three standardized cross-sectional health surveys conducted between 2004 and 2013 to describe the trends in prevalence of hypertension and its associated factors as well as the BP distributions in Guangzhou, China.

## Materials and Methods

### Study population

The Community Health Diagnosis of Guangzhou is a program to assess health status of community residents in Guangzhou, which carried out by Guangzhou Center for Disease Control and Prevention every five years. A representative sample of permanent residents (aged 15 yr or older) in Guangzhou City was recruited. Three standardized cross-sectional health surveys were conducted in 2004, 2009 and 2013.

The sample size of each survey was decided based on the Community Health Diagnosis Technical Manuals, recommended by the Department of Maternal and Child Health Care and Community Health of the Ministry of Health in China. Namely, using a family as the basic unit, 800 households were randomly sampled from community of more than 50000 people (two neighbourhoods nearby were combined if the population size of the neighbourhood was less than 50000), and single individuals were categorized as a family, a total of 9600 households. All family members were interviewed in this survey, including infants, children, adolescents, pregnant people and aged people. Participants between 15 to 80 yr old were selected except pregnant women. The sample size of each survey was 27299, 23467 and 18362 participants in 2004, 2009 and 2013. We selected this age range because BP is more susceptible to social economic factors than other age groups. The response rates for survey 2004, survey 2009 and survey 2013 were 93.1%, 98.87%, and 90.5%, respectively.

Informed consent was taken from the participants and the study was approved by Ethics Committee of the university.

### Sampling method

**Fig. 1:**
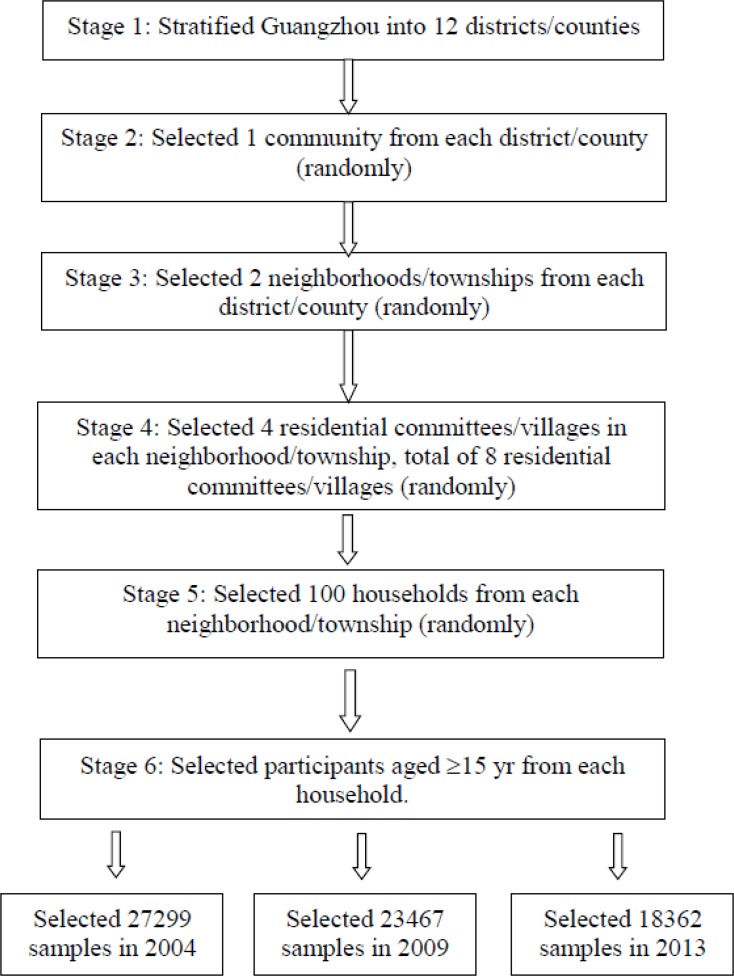
Sampling flow chart

A five-stage cluster random sampling plan was used to recruit participants in 2004, 2009 and 2013, respectively. In the first stage, one community health service center from each district was randomly selected as a sample point. Therefore, 12 locations were sampled and used for all three surveys. In the second stage, two neighborhoods (urban) or townships (rural) were randomly sampled from each district. If a community health service center had jurisdiction over only one neighbourhood, then the neighbourhood was the sample point. The third stage of sampling was conducted in each selected neighbourhood or township. Four residential committees/villages were randomly selected from each neighbourhood or township and eight residential committees/villages were directly selected from the neighbourhood when the community health service center’s jurisdiction was only one neighbourhood. In the fourth stage, 100 households were randomly sampled from each residential committee/village. A residential committee with less than 100 households was merged with another committee. In the fifth stage, all family members who live in Guangzhou for at least 6 months were selected. Samplings from first to third stage were completed by the Guangzhou Center for Disease Control and Prevention (CDC). Sampling in the fourth and fifth stage was completed by each sample point according to the principle of random sampling and was implemented with the Guangzhou CDC’s approval (Fig. 1).

### Investigation method

All participants were interviewed, completed a questionnaire and provided with a health examination at the central survey sites or households. Guangzhou CDC was responsible for developing the questionnaire. Our study chose part of the questionnaire, including demographic characteristics (e.g., name, age, sex, nationality), personal health history (common chronic disease) and lifestyle (drinking and smoking habits). If the respondents could not finish the questionnaire during the investigation process (such as mental or hearing impairment, etc.), then a person knowledgeable background could reply on their behalf. Health examinations were conducted by physicians received specific training for the health assessments. Height, weight, and BP were collected in the health examination. Height and weight were measured before breakfast in the morning while wearing light clothes and without shoes. The participants were told to avoid alcohol, coffee, tea and exercise before the BP measurement. The BP measurement was taken twice for the right arm while participants were in a sitting position, and each measurement was separated by a 5-min interval. If the difference between the two readings were greater than 6 mmHg, then a third measure was taken.

The average two or three readings (if necessary) were used for analysis. To ensure the reliability of survey data, systematic training was provided for investigators to ensure understanding of the study objective, meaning and methods. Investigation forms were checked by staff for errors or omissions after the questionnaire recovery, so that phone calls could be made to supplement the information. The replacement rate was not allowed to exceed 10%.

### Statistical analysis

Epidata (ver. 3.0) was used to establish database. All data were double inputted and checked. The frequency differences between research variables and trends were compared using the chi-squared test or trend of chi-square test. BP and age were compared using the *F* test. Age-standardised prevalence was calculated based on China's sixth census data in 2010 using the age groups 20–34 yr, 35–49 yr, 50–64 yr and 65–80 yr. Multinomial logistic regression was used to estimate potential risk factors for hypertension, including age, sex, nationality, BMI, smoking, drinking alcohol from the three surveys. Statistical analyses were conducted used SPSS 21.0 (Chicago, IL, USA). Two-tailed *P* values <0.05 were considered statistically significant. The 95% confidence intervals were calculated and presented.

## Results

Table 1 provides demographic characteristics of residents for sex, age, nationality, BMI, and BP. A total of 27299, 23467 and 18362 participants completed the survey in 2004, 2009 and 2013, respectively. Participants aged 15–34 yr and 35–49 yr showed a decreasing trend (*P*<0.001) while those aged 50–64 yr and 65–80 yr showed an increasing trend (*P*<0.001) between 2004 and 2013. Young people aged 15–34 yr decreased from 36.1% in 2004 to 24.2% in 2013, and people over age 50 yr increased from 33.1% to 47.0% during the same time. The mean ages in 2003, 2009 and 2013 were 42.8, 43.6 and 47.5 yr, respectively, which was a statistically significant increase (*P*<0.001). People with a BMI ≥24 or 28 have increased from 19.4% to 27.3% and 4.6% to 10.7% in 2004 to 2013 (*P*<0.001). The population also had a higher BP, due to an SBP increase from 117.9 to 123.2 mmHg and a DBP increase from 75.1 to 76.4 mmHg during 2004–2013 (*P*<0.001).

**Table 1:** Characteristics of participants aged 15–80 yr in Guangzhou, China, 2004–2013

***Characteristic***	***Survey 2004***	***Survey 2009***	***Survey 2013***	**P*_trend_***
All	27299	23467	18362	-
Sex, no.(%)				
Female	14258(52.2)	11834(50.4)	10022(54.6)	<0.001
Age group (y)				
15–34	9858(36.1)	7647(32.6)	4452(24.2)	<0.001
35–49	8389(30.7)	7106(30.3)	5281(28.8)	<0.001
50–64	5609(20.5)	5682(24.2)	5636(30.7)	<0.001
65–80	3443(12.6)	3032(12.9)	2993(16.3)	<0.001
Nationality				
Han population	26546(99.2)	23418(99.8)	18280(99.7)	<0.001
Body mass index (BMI, kg/m^2^)				
<18.5	5966(21.8)	2766(11.8)	1246(6.8)	<0.001
18.5–23.9	14808(54.2)	14043(59.8)	10137(55.2)	<0.001
24–27.9	5313(19.4)	5302(22.6)	5019(27.3)	<0.001
≥28	1252(4.6)	1356(5.8)	1960(10.7)	<0.001
Mean age (mean±SD, y)	42.8±16.0	43.6±16.8	47.5±16.2	<0.001
BMI, mean±SD, kg/m^2^				
All	22.4±3.3	22.4±3.4	23.1±3.4	<0.001
Male	22.5±3.1	22.6±3.3	23.3±3.4	<0.001
Female	22.3±3.4	22.2±3.6	22.9±3.5	<0.001
BP, mean±SD, mmHg								
Systolic								
All	117.9±16.8	123.4±17.5	123.2±19.6	<0.001
Male	119.9±15.6	125.5±15.7	124.7±19.3	<0.001
Female	116.2±17.6	121.4±18.8	122.0±19.8	<0.001
Diastolic								
All	75.1±9.6	77.5±10.3	76.4±12.6	<0.001
Male	76.5±9.2	78.6±9.7	77.6±11.9	<0.001
Female	73.8±9.7	76.5±10.7	75.4±13.0	<0.001

Table 2 shows the changing trends regarding hypertension prevalence. Hypertension prevalence of community residents in Guangzhou has increased between 2004 and 2009, with a moderated decline between 2009 and 2013. The age-standardised prevalence was 12.5%, 16.0% and 14.0% while crude prevalence was 14.6%, 19.1% and 18.8% in 2004, 2009 and 2013. The prevalence of residents aged 15–49 yr increased significantly from 2004–2013 (*P*<0.001), but those aged 50–80 yr increased from 2004–2009 and declined from 2009–2013. We can find a similar law after gender stratification in both age-standardized and crude rates. The hypertension prevalence increases with residents’ age, with a peak in 65–80 yr old.

**Table 2:** Trends in prevalence (%) in hypertension of participants aged 15–80 yr in Guangzhou by age and sex, 2004–2013

***Variables***	***Survey 2004***	***Survey 2009***	***Survey 2013***	***P_trend_***
All Age-standardised	12.5	16.0	14.0	-
Crude prevalence	3141(14.6)	4101(19.1)	3426(18.8)	<0.001
15–34 yr	148(2.0)	184(2.6)	128(2.9)	<0.001
35–49 yr	558(8.7)	787(12.4)	618(11.8)	<0.001
50–64 yr	1230(26.5)	1718(33.1)	1477(26.4)	0.563
65–80 yr	1205(40.2)	1412(48.9)	1203(40.5)	0.801
Male Age-standardised	12.8	16.6	15.1	-
Crude prevalence	1500(15.4)	2000(19.4)	1605(19.4)	<0.001
15–34 yr	99(3.1)	137(3.9)	92(4.5)	0.008
35–49 yr	288(9.6)	391(13.1)	336(14.1)	<0.001
50–64 yr	536(26.1)	800(33.0)	661(26.4)	0.902
65–80 yr	577(38.1)	672(47.7)	516(39.0)	0.482
Female Age-standardised	12.3	15.4	12.8	-
Crude prevalence	1641(14.0)	2088(18.8)	1821(18.3)	<0.001
15–34 yr	49(1.2)	46(1.3)	36(1.5)	0.314
35–49 yr	270(7.8)	393(11.8)	282(9.8)	0.004
50–64 yr	694(26.9)	914(33.2)	816(26.4)	0.461
65–80 yr	628(42.3)	735(49.9)	687(41.6)	0.582

**Fig. 2:**
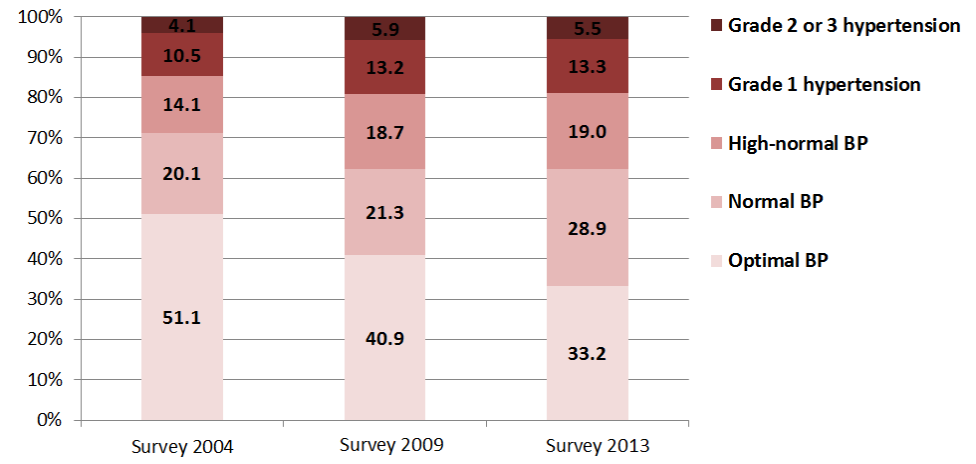
Distribution of BP in different stages of whole population in Guangzhou, China, 2004–2013

**Fig. 3:**
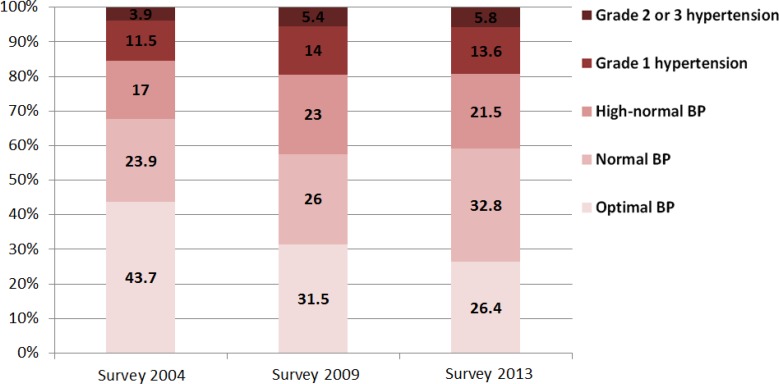
Distribution of BP in different stages of males in Guangzhou, China, 2004–2013

**Fig. 4:**
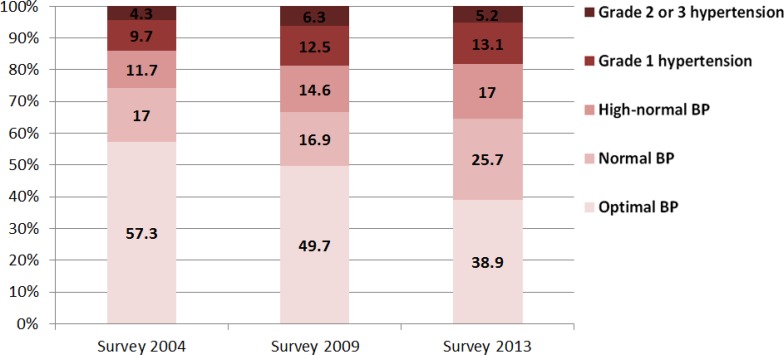
Distribution of BP in different stages of females in Guangzhou, China, 2004–2013

Figs. 2–4 shows that the proportion of residents with optimal BP in Guangzhou city declined from 51.1% in 2004 to 33.2% in 2013 while the proportion of residents with grade 1 hypertension rose from 10.5% in 2004 to 13.3% in 2013. From 2004 to 2009, the number of Guangzhou residents with high-normal hypertension (14.1% vs 18.7%) increased remarkably. From 2009 to 2013, it rose slowly (18.7% vs 19.0%). Through gender comparison, the number of females with optimal BP was greater than males, while the proportion of males was higher for high-normal hypertension (also called prehypertension) compared to females in both surveys.

Age was significantly associated with greater odds of hypertension in 2004, 2009 and 2013, the danger of suffering hypertension rises with the increasing of age (Table 3).

**Table 3:** Factors associated with hypertension among community residents in Guangzhou, 2003–2013

***Factors***	***2004***		***2009***		***2013***	
	***OR***	***95% CI***	***OR***	***95% CI***	***OR***	***95% CI***
Age group(yr)						
18–34 (Ref)						
35–49	6.16	4.52–8.40	4.98	4.04–6.14	3.51	2.80–4.41
50–64	22.53	16.66–30.49	18.44	15.14–22.46	10.58	8.52–13.13
65–80	47.00	34.59–63.87	35.24	28.40–43.74	23.62	18.69–29.85
Sex						
Female(Ref)						
Male	0.99	0.90–1.08	1.07	1.01–1.16	0.99	0.89–1.12
Nationality						
Han population(Ref)						
Minority	1.01	0.62–1.64	0.23	0.05–1.01	1.37	0.73–2.56
BMI, kg/m^2^						
18.5–23.9(Ref)						
24–27.9	2.01	1.76–2.29	2.08	1.87–2.32	2.05	1.86–2.26
≥28	4.52	3.73–5.47	4.47	3.77–5.31	3.93	3.41–4.53
Smoking												
Never(Ref)						
Ever	1.10	0.76–1.58	1.07	0.92–1.24	1.17	1.02–1.34
Drinking alcohol						
Never(Ref)						
Ever	1.34	1.18–1.53	1.37	1.21–1.56	1.20	1.07–1.36

Male (OR=1.07, 95%CI: 1.01 to 1.16) was significantly associated with greater odds of hypertension in 2009. People whose BMI ≥24 (OR_2004_=2.01, 95%CI: 1.76 to 2.29; OR_2009_=2.08, 95%CI: 1.87 to 2.32; OR_2013_=2.05, 95%CI:1.86 to 2.26) and ≥28 (OR_2004_=4.52, 95%CI:3.73 to 5.47; OR_2009_=4.47, 95%CI:3.77 to 5.31; OR_2013_=3.93, 95%CI:3.41 to 4.53) was significantly associated with greater odds of hypertension compared with those BMI ranged from 18.5 to 23.9. People ever smoked (OR=1.17, 95%CI:1.02 to 1.34) was significantly associated with greater odds of hypertension in 2013 compared with those never smoked.

People ever drank alcohol (OR_2004_=1.34, 95%CI:1.18 to 1.53; OR_2009_=1.37, 95%CI:1.21 to 1.56; OR_2013_=1.20, 95%CI:1.07 to 1.36) was significantly associated with greater odds of hypertension compared with those never drank alcohol in both 2004, 2009 and 2013.

## Discussion

There was an overall increase of age-standardised prevalence of hypertension in 2004–2009 (12.5% vs 16.0%), and a slight decline in 2009–2013 (16.0% vs 14.0%), which was more obvious in individuals aged 50 yr or above. Studies of different magnitudes and different research groups demonstrated varying prevalence (12, 13). Overall, there was a rapidly rising prevalence of hypertension in China in the recent ten years (14). Guangzhou is the largest and most prosperous trading city in southern China and has the urbanization rate of 85.43% in 2014. This study used three standardised cross-sectional health surveys to describe the trends in prevalence of hypertension and its associated factors as well as the BP distributions in Guangzhou, China.

Two Chinese national hypertension surveys (4) report the prevalence of hypertension at 13.6% in 1991, 18.8% in 2002, which were similar to our study. Hypertension prevalence in this study (≥140 mm Hg SBP and/or ≥90 mm Hg DBP) was lower than Canada (19.5%), USA (29%), Germany (30% to 32%) and England (30%) during 2006 to 2011 (15, 16). The prevalence of hypertension in the USA declined from 29.7% to 20.4% between 1960–1991 according to the NHANES survey (17, 18). Overall, hypertension showed a downward trend in some developed countries but an upward trend in developing countries (17–19). Over the past decade, there has been a declining trend (*P*<0.0001) in central China but a rising trend (*P*<0.0001) in other regions (14), but our study showed a slight decline in Guangzhou. This may be due to the continuous improvement of medical standards as well as residents' awareness and control of hypertension in Guangzhou. Data provided by Guangdong Provincial Center for Disease Control and Prevention (20) indicated that the awareness of hypertension of Guangdong residents from 2002 to 2010 rose from 15.1% to 25.2%, and the treatment rate increased from 11.8% to 17.7%. Besides, Guangzhou’s air quality improved year by year, the rate of up-to-standard (PM2.5 <100) air quality has reached 95.07% and 97.81% between 2009–2010 (21), which is more suitable for residents outdoor activities than other big cities in China.

The prevalence of hypertension in China demonstrates a high rate in the north and a low rate in the south, the salt intake of residents in northern China appears to be higher compared to the south (22). On the other hand, with annual average temperature reaches 22 degrees in Guangzhou, exercise is year-round attractions in Guangzhou because of the warm weather. Nevertheless, the hypertension prevalence trend in Guangzhou, a city located in the supposed low-incidence area, does not provide optimism. The substantial rise in the past 10 yr requires adequate attention. As a developing country, China is characterized by a lower prevalence of hypertension than some developed countries, but its large population base makes results in a severe disease burden despite the seemingly low rate. Therefore, rather than wait to intervene only after the established hypertension peak, we should learn lessons from the successful experience of developed countries in controlling hypertension before the peak prevalence to effectively curb the increasing trend of hypertension.

People falling into the high-normal BP range account for 14.1% of the population of Guangzhou in 2004 and rose to 19.0% in 2013, representing a group highly susceptible to hypertension. This new trend has pointed out the direction of hypertension prevention and control. Apart from the patients themselves, the population with high-normal BP should be brought into focus. In 2010, Guangzhou called for exercise breaks in the daytime so that employees in government agencies, companies and offices could have some physical training during the work (23). Besides, recent bike share initiatives have increased to a certain extent the amount of physical activity of the youth. In the future, Guangzhou will release tens of thousands of bikes for public use as a way to expand the choices of citizens for short trips.

Our study showed that both average age and weight had increased from 2004–2013, which may be one of the reasons for growing hypertension. Multivariate analysis found that age was the most important influencing factor for the hypertension prevalence in all three surveys conducted at different times and showed that the older the age, the greater the risk of hypertension. Comparison of different ages indicated that the OR value of residents of all ages that suffer from hypertension declined in each successive survey. For example, OR value for ages 65–80 yr was 47.0 in 2004, 35.2 in 2009, and 23.6 in 2013. Clearly, the effect of age in the process decreased at each survey, especially in those age > 50 yr old. The elderly are more health-conscious and they have more time after retirement to take physical exercise, such as badminton playing, dancing in public places, walking and jogging. For the young generations, however, reduction is less significant, probably due to the pressure from work and life and the lack of time for exercise. Previous studies have found that age and high BMI were potential risk factors for hypertension control (24, 25), which was similar to our conclusion.

China is one of the fastest growing countries all over the world during the survey period. Residents were more likely to carry some lifestyle factors such as changes in dietary, insufficient physical activity and overeating, which could be the important cause leading to the obesity. Previous study (26, 27) involved 13000 males found that past smokers and current smokers both had the risk of hypertension compared with never smokers, and smoking cessation was connected with lower BP and minor hypertension risk. Our study did not distinguish between past smokers and current smokers, but we support the point to quit smoking or stop smoking. Besides, this large study also showed that the association between drinking alcohol and the risk of hypertension among community residents in Guangzhou tends to be proportional to the alcohol drinking of hypertension. Some studies have discovered that alcohol consumption is associated with the development of hypertension, and estimated that approximately 5% to 30% of hypertension can be attributed to alcohol (28, 29). Limiting alcohol consumption is an important strategy to reduce or prevent growing hypertension in China.

Of course, there are certain limitations to our study. One is that a cross-sectional study generates inevitable recall bias. Moreover, despite revealing differences in prevalence between ages, cross-sectional studies fail to reflect the separate age effect. The age–period–cohort (APC) model, recommended for further study, can be used to distinguish the separate age effect regarding the hypertension prevalence in continuous cross-section data. Furthermore, there are many factors affecting hypertension prevalence such as dietary habits, lifestyle, public resources, economics and health care (30), and this may exhibit a complex interplay in these studies (31). Finally, it addressed the Chinese Han population majority (99%), hence it should be careful to draw a conclusion in other ethnic.

## Conclusion

Hypertension prevalence of community residents in Guangzhou has increased between 2004 and 2009, with a moderated decline between 2009 and 2013. Age, BMI, sex, smoking and drinking alcohol were the influential factors for hypertension. With the urban and economic development of Guangzhou, hypertension prevalence will still increase. Public health strategies should be still focused on the aging population and hypertensive patients in Guangzhou. Meanwhile, we should also keep one eye on high-normal blood pressure population and to prevent them from turning into hypertensive patients.

## Ethical considerations

Ethical issues (Including plagiarism, informed Consent, misconduct, data fabrication and/or falsification, double publication and /or submission, redundancy, etc) have been completely observed by the authors.
